# Norepinephrine Inhibits Synovial Adipose Stem Cell Chondrogenesis via α2a-Adrenoceptor-Mediated ERK1/2 Activation

**DOI:** 10.3390/ijms20133127

**Published:** 2019-06-26

**Authors:** Karima El Bagdadi, Frank Zaucke, Andrea Meurer, Rainer H. Straub, Zsuzsa Jenei-Lanzl

**Affiliations:** 1 Dr. Rolf M. Schwiete Research Unit for Osteoarthritis, Orthopedic University Hospital Friedrichsheim gGmbH, 60528 Frankfurt/Main, Germany; 2 Laboratory of Experimental Rheumatology and Neuroendocrine Immunology, Department of Internal Medicine, University Hospital Regensburg, 93053 Regensburg, Germany

**Keywords:** synovial adipose stem cells, sympathicus, norepinephrine, adrenoceptors, chondrogenesis, osteoarthritis, physioxia

## Abstract

In recent years, first evidences emerged that sympathetic neurotransmitters influence osteoarthritis (OA) manifestation. Joint-resident stem cells might contribute to cartilage repair, however, their chondrogenic function is reduced. The neurotransmitter norepinephrine (NE) was detected in the synovial fluid of trauma and OA patients. Therefore, the aim of this study was to analyse how NE influences the chondrogenesis of synovial adipose tissue-derived stem cells (sASCs). sASCs were isolated from knee-OA patients synovia. After adrenoceptor (AR) expression analysis, proliferation and chondrogenic differentiation in presence of NE and/or α- and β-AR antagonist were investigated. Cell count, viability, chondrogenic and hypertophic gene expression, sulfated glycosaminoglycan (sGAG) and type II collagen content were determined. Key AR-dependent signaling (ERK1/2, PKA) was analyzed via western blot. sASC expressed α1A-, α1B-, α2A-, α2B-, α2C-, and β2-AR in monolayer and pellet culture. NE did not affect proliferation and viability, but 10^−7^ and 10^−6^ M NE significantly reduced sGAG and type II collagen content as well as ERK1/2 phosphorylation. These effects were fully reversed by yohimbine (α2-AR antagonist). Our study confirms the important role of NE in sASC chondrogenic function and provides new insights in OA pathophysiology. Future studies might help to develop novel therapeutic options targeting neuroendocrine pathways for OA treatment.

## 1. Introduction

Osteoarthritis (OA) is the most common degenerative joint disorder affecting about 10% of the global population [1]. OA is characterized by progressive degradation of articular cartilage [2] leading to pain, stiffness, and disabilities. There is no proven treatment for OA at present, therapies target late stage pain or inflammation, but in most cases prosthetic joint replacement remains as final therapy option [3]. OA pathogenesis involves the interaction of cartilage-surrounding joint tissues including synovial tissue, bone, ligaments, meniscus, and the synovial fluid [4,5,6]. Since articular cartilage is avascular, aneural, and alymphatic chondrocytes exhibit a very limited self-regenerative capacity [7]. However, during the past decades, the existence of highly regenerative mesenchymal stem cells (MSCs) was confirmed in cartilage-surrounding tissues including synovium, bone marrow, and synovial fluid [8,9]. These cells might migrate to damaged cartilage areas and differentiate to chondrocytes [9].

There is strong evidence that synovial adipose tissue-derived MSCs (sASC) contribute to the repair of cartilage injuries [10,11]. However, the chondrogenic capacity of MSCs detected in OA cartilage is insufficient [10] and the reasons for this are not completely understood. Recent studies demonstrated that peripheral sympathetic nerve fibers are involved in chondrogenic extracellular matrix (ECM) deposition during endochondral ossification [12,13,14]. Furthermore, sympathetic nerve fibers expressing tyrosine hydroxylase (TH), which is the key enzyme of catecholamine biosynthesis, have been detected in healthy and OA joint tissues [15,16,17]. TH-positive nerve fibers release high concentrations of norepinephrine (NE) into the synovial fluid as shown in previous studies [18].

NE is one major catecholaminergic neurotransmitter of the sympathetic nervous system and binds to specific adrenergic receptors (AR) depending on its concentration [19,20]. All α and β adrenergic receptors subtypes (α1A, α1B, α1D, α2A, α2B, α2C, β1, β2, β3) belong to the G protein-coupled receptor family. Depending on the activation of alpha subunits of ARs (Gs, Gi, or Gq), different intracellular signaling pathways become activated [21]. At low concentrations (≤10^−7^ M), NE mainly acts via α-ARs with subsequent protein kinase a (PKA) and cAMP inhibition (Gαi signaling). In contrast, NE at high concentrations (≥10^−7^ M) preferentially acts via β-ARs leading to PKA and cAMP increase (Gαi signaling). Furthermore, β-arrestin regulates AR-mediated signaling by binding to phosphorylated ARs which in turn leads to the alternative ERK1/2 pathway [22,23,24]. Previous studies investigated the effects of NE on articular cartilage and described that NE induced catabolic effects in chondrocytes by inhibiting type II collagen synthesis via β2-AR or accelerated cartilage ECM degradation via α2A-AR suggesting that mainly the α2A- and β2-AR subtypes might play a role in disturbed cartilage regeneration during OA development [25,26,27]. Recently, we demonstrated that bone marrow-derived MSCs (BMSCs) obtained from trauma patients express α- and β-ARs [18] and that NE inhibits chondrogenic differentiation with concomitant induction of hypertrophy by the β2-AR under normoxic conditions (20% O_2_) [18]. However, no studies using sASC regarding NE sensitivity and chondrogenic potential under NE influence have been performed until now. Furthermore, most existing studies on the regeneration potential of MSCs have been performed under normoxia, although cartilage microenvironment contains only about 2% O_2_ representing the “physioxic condition” [28,29]. Therefore, the aim of the present study was to address the effect of NE on sASC proliferation and chondrogenic differentiation under physioxic conditions.

## 2. Results

### 2.1. Stem Cell-Specific Surface Markers and Chondrogenic Differentiation Potential of ASCs

**Figure 1:**
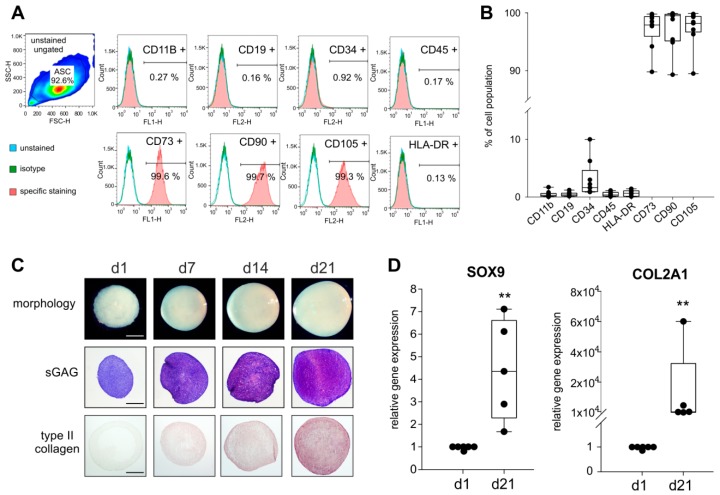
Analysis of mesenchymal stem cells (MSC)-specific surface markers and chondrogenic differentiation capacity of synovial adipose tissue-derived MSCs (sASC) obtained from osteoarthritis (OA) patients. (**A**) Human sASCs derived from OA patients were positive for CD73, CD90, and CD105 and were negative for CD11b, CD19, CD34, CD45 and human leukocyte antigen–DR isotype (HLA-DR) using FACS analysis (blue line—unstained negative control, green line—isotype control, red line—the target surface marker). (**B**) Quantification of MSC-specific marker expression on sASCs from eight different OA patients (*n* = 8). Data are presented as box plots with the 10th, 25th, 50th (median), 75th, and 90th percentiles. Each black circle represents an individual patient: (**C**) Macroscopic, histologic (sGAG) and immunohistochemical (type II collagen) analysis of untreated chondrogenic sASC pellets at day1, day 7, day 14, and day 21 of differentiation (bars, 500 μm). (**D**) Relative gene expression of SOX9 and type II collagen in untreated ASC pellets after 21 days of chondrogenesis. Data are presented as box plots with the 10th, 25th, 50th (median), 75th, and 90th percentiles. Each black circle represents an individual patient (*n* = 5–6). Significant *p*-values against untreated control are presented as ** = *p* < 0.01.

Stem cell-specific surface markers of ASCs were investigated by FACS analysis of CD11b, CD19, CD34, and HLA-DR. More than 90% of the ASCs were positive for CD73, CD90, and CD105 as well as negative for HLA-DR, CD11b, CD19, CD34, and CD45 as suggested by The Mesenchymal and Tissue Stem Cell Committee of the International Society for Cellular Therapy [28] (Figure 1A,B).

The macroscopic observation revealed a well-condensated nodular morphology with increasing pellet size from day 1 until day 21 and the histological analysis by the metachromatic DMMB staining confirmed a characteristic and increasing sGAG deposition during 21 days of chondrogenesis. Similarly, type II collagen staining intensity steadily increased from day 1 to day 21 (Figure 1C). Expression of SOX9 and COL2A1, the two major marker genes indicative of chondrogenic differentiation, increased significantly from day 1 until day 21 (both SOX9 (~4-times) and COL2A1 (~10^4^-times) day 21 to day 1 *p* = 0.008; Figure 1D).

### 2.2. AR Expression Profile of ASCs in Monolayer and Pellet Cultures

**Figure 2:**
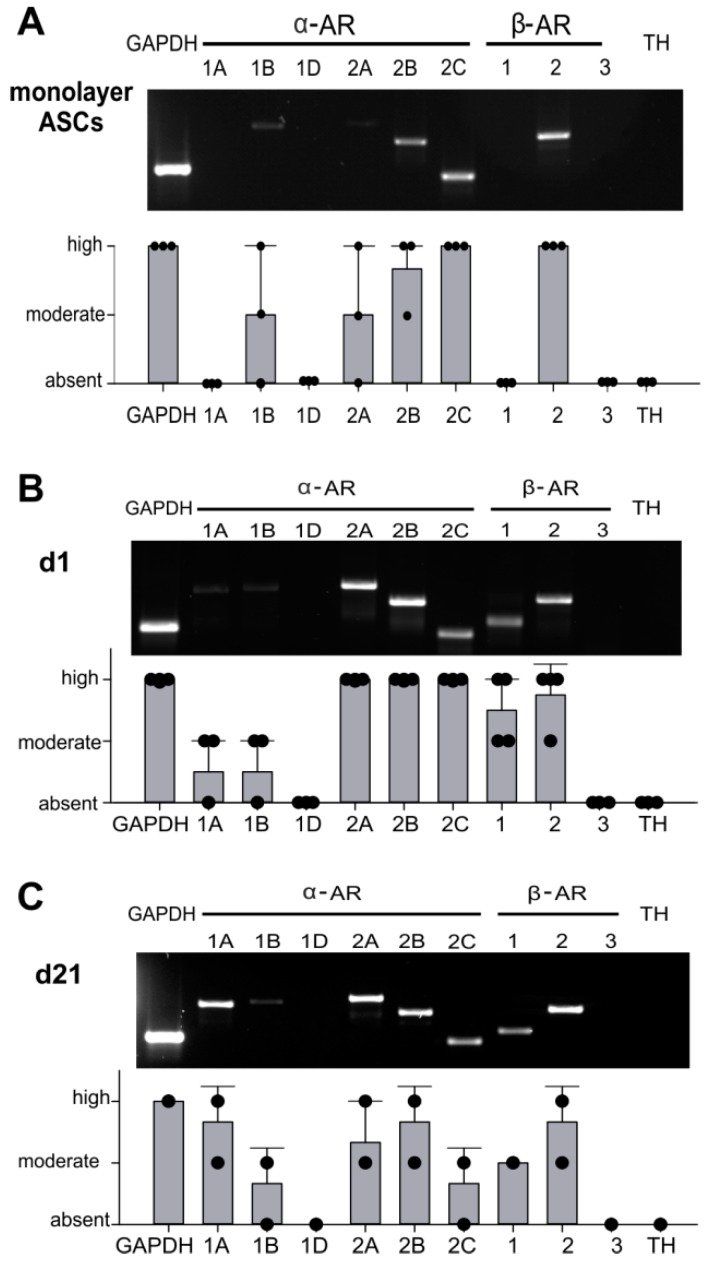
Adrenoceptor (AR) and tyrosine hydroxylase (TH) gene expression of sASCs in monolayer and pellet cultures (**A**) RT-PCR of AR and TH gene expression profile in untreated sASC in monolayer culture and average score of AR and TH gene expression in sASCs from three different OA patients. (*n* = 3, mean ± standard deviation). (**B**) AR and TH gene expression profile in untreated sASC in three-dimensional chondrogenic pellet culture at day 1 and average score of AR and TH gene expression in sASCs from four different OA patients. (*n* = 4, mean ± standard deviation). (**C**) AR and TH gene expression profile in untreated sASC in three-dimensional chondrogenic pellet culture at day 21 of differentiation and average score of AR and TH gene expression in sASCs from four different OA patients. (*n* = 4, mean ± standard deviation).

**Figure 3:**
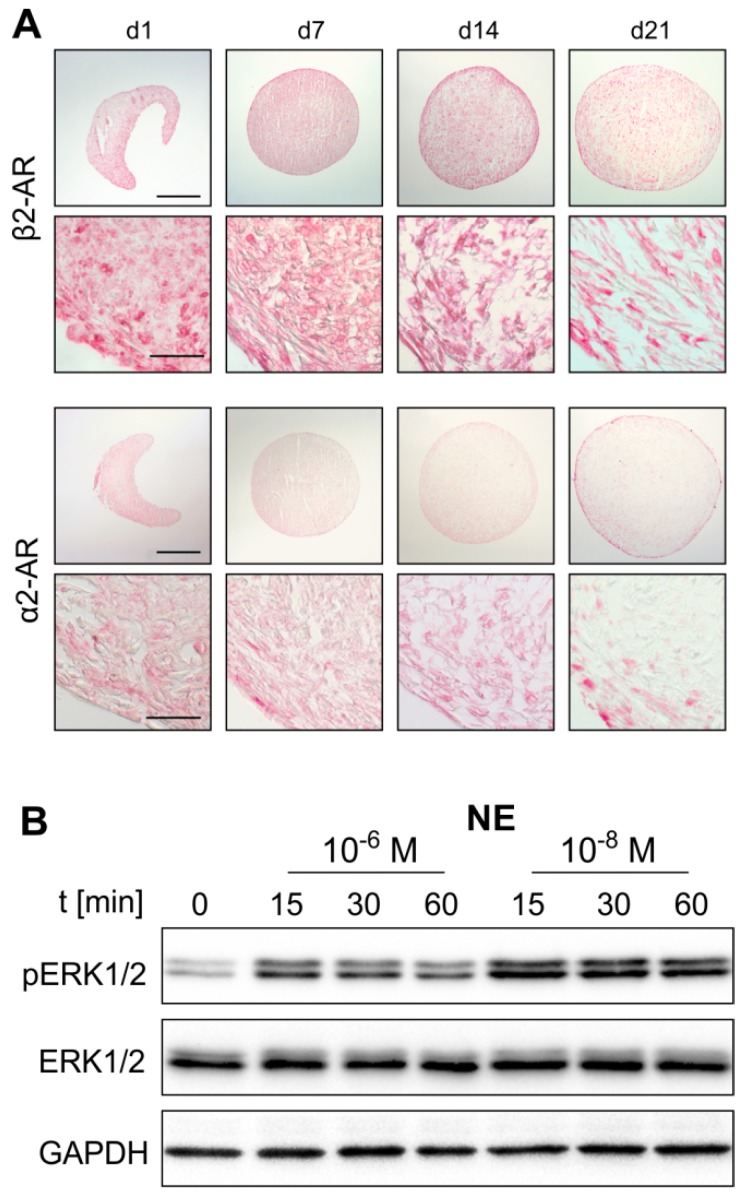
AR protein expression during sASC chondrogenesis and norepinephrine (NE)-mediated activation of intracellular signaling. (**A**) Immunohistochemical analysis of β2-AR and α2a-AR during sASC chondrogenesis on days 1, 7, 14, and 21 of differentiation (bars, 500 μm in upper panels and 50 μm in lower panels). (**B**) Western blot analysis of total ERK1/2 as well as ERK1/2 phosphorylation in chondrogenic sASC culture in the absence or presence of NE (10^−8^ M and 10^−6^ M, representative blot of one OA patient).

First, the expression of different AR subtypes was screened by RT-PCR. Monolayer ASCs expressed α1B, α2A-, α2B-, α2C-, and β2-AR. Gene expression levels of α2C- and β2-AR were the highest. The receptors α1B-AR, α2A-AR, and α2B-AR were moderately expressed. The receptors α1A-, α1D-, β1-AR and TH could not be detected (Figure 2A). Chondrogenic sASCs in pellets expressed α1A-, α1B-, α2A-, α2B-, α2C-, β1-and β2-AR, at day 1 and also at day 21 of chondrogenesis (Figure 2B,C). The average gene expression level of these ARs was similar at both time points except for strongly increasing α1A-AR expression from day 1 until day 21. The ARS α1D-AR, β3-AR and TH were not detectable at the gene expression level (Figure 2B,C). Next, the two most prominent ARs with opposing downstream signaling pathways, α2A-AR and β2-AR, were stained immunohistochemically in order to confirm their expression also at the functional protein level. Both, α2A-AR and β2-AR were detected at each time point during chondrogenesis (day 1, 7, 14, and 21) (Figure 3A). No age-, gender-, or medication-dependent differences were observed regarding AR expression neither in monolayer nor in pellet cultures. Furthermore, NE stability in the cell culture medium over 24 h was tested in order to clarify which NE concentration is present or might act during chondrogenesis with media change every other day. After one day in the incubator, about 40–50% of the initial NE concentration was measurable, which was independent of the applied GSH amount (Supplementary Figure S1).

### 2.3. NE-Mediated Intracellular Signaling

Western Blot analyses of monolayer sASCs revealed that NE treatment results in ERK1/2 phosphorylation. Both, 10^−8^ and 10^−6^ M NE concentrations activated the ERK1/2 pathway already after 15 min (Figure 3B). Furthermore, while the ERK1/2 phosphorylation increased dose-dependently by NE treatment (*p* = 0.02 for NE 10^−6^ M), the PKA pathway was not activated (Supplementary Figure S2).

### 2.4. Effect of NE on ASC Proliferation

**Figure 4:**
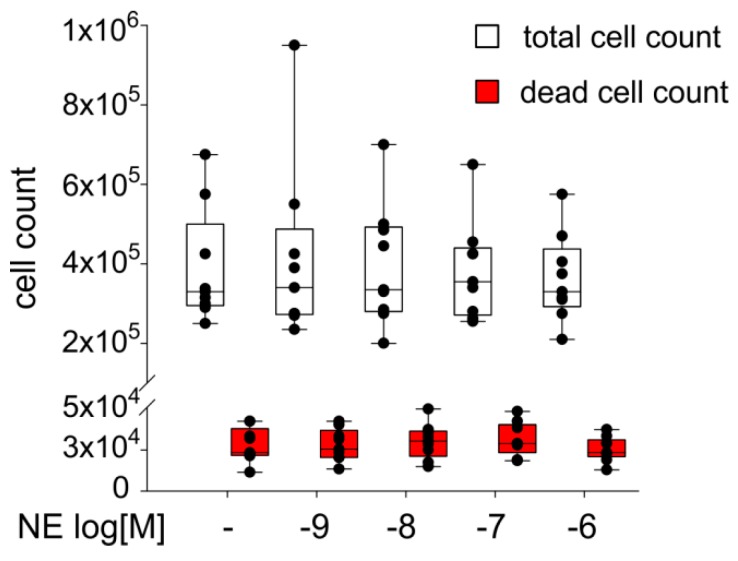
Proliferation capacity of sASCs in presence of NE in different concentrations (10^−9^–10^−6^ M). The white boxes represent the total number of cells, the red boxes represent the number of dead cells after seven days. Data are presented as box plots, where the boxes represent the 25th to 75th percentiles, the lines within the boxes represent the median, and the lines outside the boxes represent the 10th and 90th percentiles (untreated control = 100%, broken line). Each black circle represents an individual patient (*n* = 8).

The sACSs were treated for seven days with NE (10^−9^–10^−6^ M). When compared to the untreated control group, treatment with NE at different concentrations had no significant effect on the cell count. Also the number of dead cells was not influenced by NE (Figure 4). Similarly, measurement of ASC viability after NE treatment by LDH-assay revealed no changes compared to control (Supplementary Figure S3A).

### 2.5. Effects of NE on sASC Chondrogenesis

**Figure 5:**
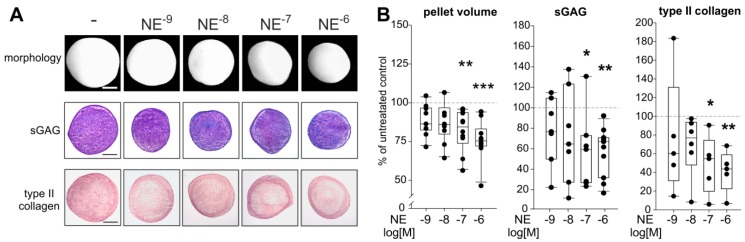
Effect of NE on sASC chondrogenesis. (**A**) Macroscopic, and histologic (sGAG) analysis as well as immunohistochemical type II collagen staining of untreated or NE-treated (10^−9^–10^−6^ M) chondrogenic sASC pellets at day 21 of differentiation (bars 500 μm). (**B**) Quantification of pellet volume and biochemical quantification of type II collagen and sGAG content of chondrogenic sASC pellets in the absence or presence of NE (10^−9^–10^−6^ M) at day 21. Each circle shows the mean of 3 replicates per patient (*n* = 6–10). Values are the percent of control (control = 100%, dashed line). Box plots are explained in legend to Figure 1. Significant *p*-values against untreated control are presented as * *p* < 0.05, ** *p* < 0.01, *** *p* < 0.001.

NE concentrations used had no proliferative, cytotoxic, or apoptotic effects during chondrogenesis as reflected by LDH measurements and dsDNA quantification (Supplementary Figure S3B,C). The morphological examination revealed that chondrogenic pellets treated with NE (10^−9^–10^−6^ M) were smaller than untreated pellets at day 21 (Figure 5A). This observation was confirmed by the calculation of pellet volumina: the pellet volume at day 21 was significantly reduced after incubation with 10^−6^ M (*p* < 0.001) as well as with 10^−7^ M NE (*p* = 0.007) (Figure 5B). 

The reduction in pellet volume was accompanied by less blue-to-purple color change of the metachromatic dye DMMB, indicating a lower amount of sGAGs in NE-treated pellets (Figure 5A). The biochemical quantification confirmed this observation, because 10^−7^ and 10^−6^ M NE treatment resulted in significantly lower sGAG concentrations compared to untreated control group (Figure 5B; *p* = 0.04 for 10^−7^ M NE and *p* = 0.006 for 10^−6^ M NE). In addition, type II collagen staining intensity was reduced in NE-treated pellets at day 21 as shown by immunohistochemistry (Figure 5A). This finding was confirmed by type II collagen ELISA showing significantly reduced type II collagen amounts in pellets after incubation with 10^−7^ (*p* = 0.02) and 10^−6^ M (*p* = 0.009) NE (Figure 5B). The observed effects on pellet size and ECM deposition became apparent first after day 14 of chondrogenesis. At day 7 or 14, no differences between treated and untreated groups were detected (Supplementary Figure S4).

In addition, in order to explore effects on fibrocartilaginous (COL1A1) and hypertrophic (COL10A1, RUNX2, MMP13) differentiation and ECM degeneration specific marker genes were analyzed. However, none of these genes was significantly influenced by NE treatment after 21 days (Supplementary Figure S5). Regarding sACS chondrogenesis under NE influence, no influence of age, gender, and medication was detected.

### 2.6. Reversal of NE-Mediated Effects by Specific AR Antagonists

To analyze which AR subtypes are responsible for the observed effects of NE, sASC pellets were treated with specific AR antagonists or with NE (10^−6^ M) in combination with antagonists. The treatment with NE in combination with antagonists or with antagonists alone had no proliferative, cytotoxic, or apoptotic effects during chondrogenesis as reflected by LDH measurements and dsDNA quantification (Supplementary Figure S7A,B). 

**Figure 6:**
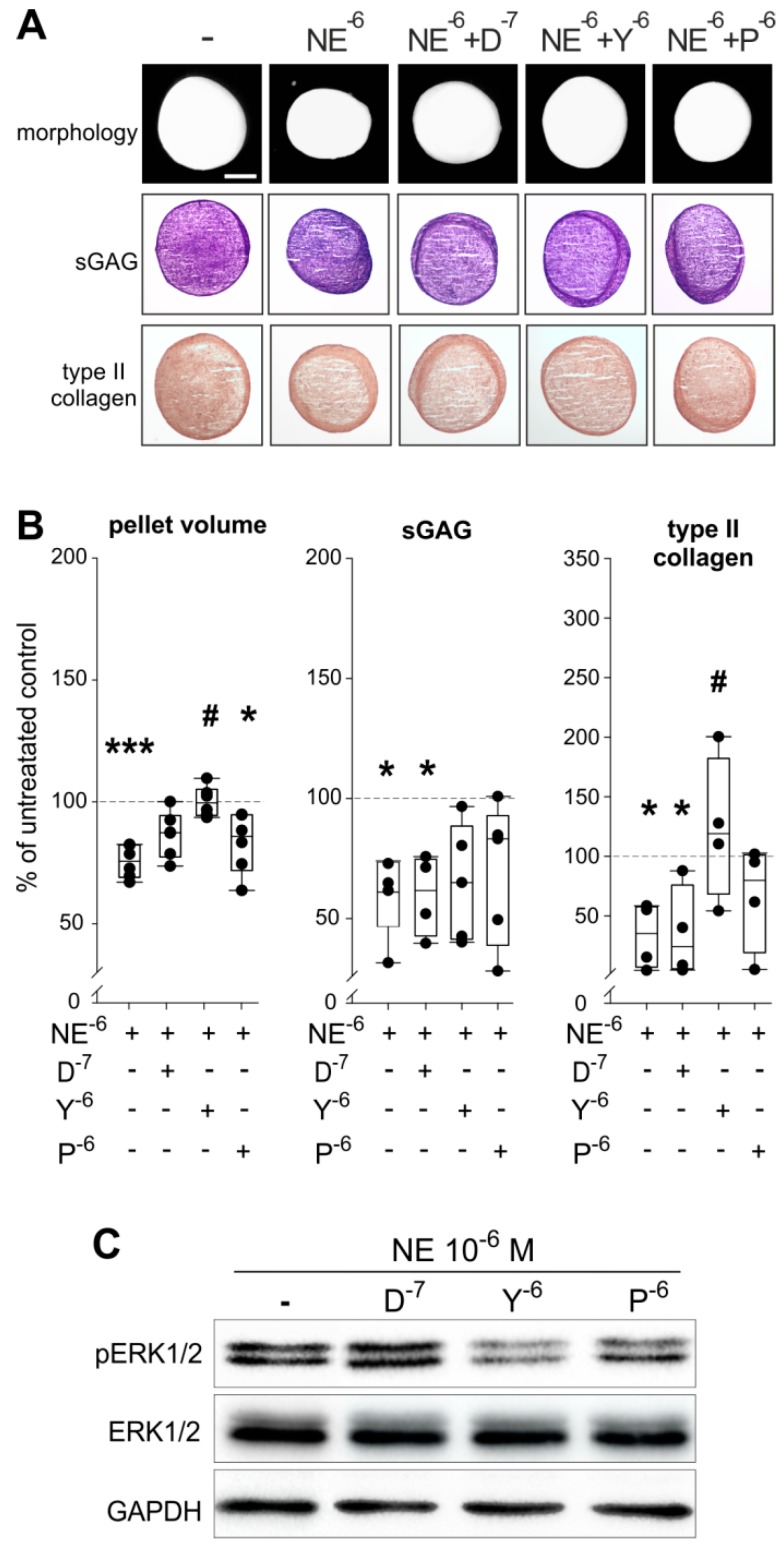
Effect of specific AR antagonists on NE effects during sASC chondrogenesis. (**A**) Macroscopic, histologic (sGAG) analysis and immunohistochemical type II collagen staining of chondrogenic sASC pellets treated with NE (10^−6^ M) or with NE plus specific AR antagonists at day 21 of differentiation (bars 500 μm). (**B**) Quantification of pellet volume and biochemical quantification of type II collagen and sGAG content of chondrogenic sASC pellets treated with NE (10^−6^ M) or with NE plus specific AR antagonists at day 21. Each circle shows the mean of 3 replicates per patient (*n* = 6–10). Values are the percent of control (control = 100%, dashed line). Significant *p*-values against untreated control are presented as * *p* < 0.05, *** *p* < 0.001; ^#^ *p* < 0.05 to NE-treatment. (**C**) Western blot analysis of total ERK1/2 and ERK1/2 phosphorylation in of chondrogenic sASC pellets treated with NE (10^−6^ M) or with NE plus specific AR antagonists: representative blot of one OA patient. Abbreviations: D—doxazosin, Y—yohimbine, P—propranolol.

The macroscopic and histological analyses revealed that the α2-AR antagonist yohimbine is able to reverse the effects of NE on pellet size and partly on type II collagen synthesis, while no effects on sGAG staining were observed by any treatment (Figure 6A). The reversing effects of yohimbine on NE-mediated reduction of pellet size and type II collagen protein amount were confirmed by quantification of pellet volumina and by type II collagen protein ELISA (Figure 6B; pellet volume: *p* < 0.001 for NE 10^−6^ M vs. control, *p* = 0.04 for *p* vs. control, *p* = 0.034 for NE 10^−6^ M vs. Y; sGAG: *p* = 0.04 for NE 10^−6^ M vs. control, *p* = 0.048 for D vs. control; type II collagen: *p* = 0.028 for NE 10^−6^ M vs. control, *p* = 0.028 for D vs. control, *p* = 0.04 for NE 10^−6^ M vs. Y). 

In contrast, the α1-AR and β2-AR antagonists (doxazosin and propranolol) were not able to modulate the observed NE-mediated effects on pellet size or on type II collagen deposition and none of the antagonists could significantly reverse the inhibitory effect of NE on sGAG (Figure 6A,B). Analysis of the ERK1/2 pathway by western blot confirmed the involvement of α2-AR in the NE-mediated effects, because the α2-AR antagonist yohimbine reversed the NE-induced phosphorylation of ERK1/2 (Figure 6C). The β2-AR antagonist propranolol only partly reversed ERK1/2 phosphorylation.

## 3. Discussion

Cartilage injuries as well as OA-associated cartilage degeneration still represent a huge orthopedic challenge [30]. Joint resident MSCs have been shown to be involved in cartilage regeneration processes [31], however, these MSCs exhibit an insufficient chondrogenic potential, although their number is increased in OA articular cartilage tissue [32]. The reasons for this contradictory phenomenon are not completely understood. Recent studies demonstrated that TH-positive nerve fibers are present in trauma and OA synovium [16,33]. Moreover, a considerable amount of the sympathetic neurotransmitter NE was detected in synovial fluid samples of trauma and OA patients [18], which might influence the regenerative capacity of the stem cells migrating from the synovial tissue to damaged cartilage areas [9]. The present study is the first demonstrating noradrenergic effects on sASC chondrogenesis and accordingly on articular cartilage homeostasis under physioxic conditions.

The initial step of this study was to confirm the molecular MSC characteristics of the isolated sASCs. Analysis of multiple independent samples over months indicated that our isolation and cell culture method resulted in homogenous sASC populations expressing the MSC-specific surface marker panel [34]. Next, the chondrogenic differentiation capacity of sASCs derived from late OA patients was analyzed. We demonstrated in this study for the first time that these sASCs condensated to typical chondrogenic pellets and synthesized considerable amounts of cartilage-specific ECM over 21 days under physioxia.

Another important prerequisite for the present study investigating the effects of NE on sASCs function was the analysis of the complete AR profile of these cells. We could show for the first time that sASCs expressed α1B, α2A-, α2B-, α2C-, and β2-AR. Kotova et al. also detected α1B, α2A-, and β2-AR, but not α2B- and α2C on abdominal subcutaneous adipose tissue-derived MSCs [35]. Interestingly, in the same tissue but in another study α1A, α1B-, α2A-, α2B-, and β1-AR were strongly but β2-AR only weakly expressed at mRNA and protein level [36]. One possible reason for this apparent discrepancy might be the tissue-specific expression of different AR subtypes. In addition, the pathophysiological situation caused by a catabolic microenvironment with increased NE concentrations might influence the expression level of ARs [36,37]. However, it was not possible to compare our sASCs derived from OA patients with ASCs from healthy or knee trauma donors, first, because healthy synovial samples were not available and second, because in most cases neither healthy nor knee trauma synovial tissue samples are surrounded or overgrown by adipose tissue in a similar manner to the OA synovium. In order to consider possible autocrine effects, the expression of TH, the key enzyme of catecholamine biosynthesis, was analyzed in sACSs. However, TH expression was not detectable, neither in monolayer nor in chondrogenic sASC cultures. Thus, autocrine effects can be excluded. This result is in line with our previous study demonstrating that BMSCs derived from knee trauma patients are TH-negative [18]. The expression of ARs during chondrogenesis was also an important point in this study. At the mRNA level α2A-, α2B-, and β2-AR were strongly expressed both at the beginning and at the end of differentiation enabling NE to unfold its effects continuously over 21 days, while α1A-, α1B-, α2C-, and β1-AR expression was lower suggesting that chondrogenesis itself does not influence AR expression. A similar AR expression profile in OA articular chondrocytes was recently described by Speichert et al. and by Lorenz et al. [26,37] indicating that the AR profile of cells from the same embryonal mesodermal origin might be conserved [38]. After detecting the major ARs, we demonstrated that these receptors activate the downstream intracellular ERK1/2 signaling pathway, without influencing the alternative PKA pathway, which is in line with previous studies investigating murine chondrocytes under NE influence [25].

Even though sASCs express different ARs, NE had no effect on the proliferation and also their viability was not influenced indicating that even highly-concentrated NE is not toxic to sASCs. One possible explanation for proliferative non-responsiveness of sASCs to NE might be the immediate vicinity to a catabolic and inflammatory microenvironment in the OA-affected joint. A similar non-responsiveness to NE was also observed by Lorenz et al. in monolayer OA chondrocyte proliferation experiments [26,39].

The relationship between NE concentration and chondrogenesis was investigated as a next step. These experiments revealed a clear inhibition of chondrogenesis when NE was added in high concentrations suggesting the involvement of β-ARs. We observed that pellets treated with NE were smaller in size. For this reduction of pellet size different processes can be responsible: First, cell apoptosis could result in smaller pellets. However, we demonstrated that cell viability was not affected by any treatment as indicated by an unchanged LDH activity. Furthermore, the cell number was constant in all pellets reflected by equal dsDNA content. Second, the pellet size might be reduced as a result of suppressed ECM synthesis and indeed, we evidenced significantly lower sGAG and type II collagen concentrations in pellets treated with NE. This finding is in line with earlier studies demonstrating decreased sGAG and type II collagen synthesis during BMSC or chondrocyte differentiation under NE influence [18,27]. The third possible explanation of reduced pellet size might be the acceleration of hypertrophy or the increase of matrix-degrading enzyme expression. However, in contrast to NE-treated BMSC pellets, hypertrophic differentiation characterized by increased COL10A1, RUNX2, and MMP13 expression was not observed in the present study [18]. One reason for suppressed hypertrophy might be the incubation of the pellets under physioxia as recently described by others [29,40].

In order to confirm the involvement of the β2-AR, the only β-AR expressed by chondrogenic pellets, specific AR antagonists were tested in combination with NE. Based on previous studies [18,27], we expected that the β2-AR antagonist propranolol might reverse the NE-mediated inhibition of sASC chondrogenesis. However, propranolol was only partly able to neutralize NE effects, while the α2-AR antagonist yohimbine significantly abrogated the inhibitory effect of NE on the pellet volume and type II collagen protein. The only existing study analyzing alpha-adrenergic signaling in chondrocytes was performed by Jiao et. al. identifying α2a-AR as responsible AR for promoting degenerative remodeling in the temporomandibular joint by induction of catabolic activities in chondrocytes [25]. Typically, α2a-AR used to be targeted by lower NE concentrations such as 10^−8^ M [12], but in the present study the effects of high NE concentrations were neutralized by α2a-AR and not or only partly by β2-AR. The reason for these mixed α2-/β2-AR-mediated effects might be the instability of NE under cell culture conditions, as demonstrated. After one or two days in culture medium, less than 40% of the initial NE is still available, thus, the 10^−7^ M initial NE concentration might decrease to 10^−8^ M. Another possible explanation would be the switch of β2-AR from Gαs to Gαi signaling as described previously by us [41]. The fact that the α1-ARs were not involved in any effect was not surprising, since no studies describing α1-AR-induced influences on chondrogenesis exist.

In conclusion, this study demonstrated that sASCs obtained from OA patients exhibit a strongly decreased chondrogenic capacity in the presence of NE mediated by α2a-dependent ERK1/2 phosphorylation and reversed by the specific α2-AR antagonist yohimbine. Thus, NE might suppress sASC-dependent regeneration of articular cartilage and contribute to the manifestation of OA. Therefore, the inhibition of α2a-adrenergic signaling pathways represents a promising approach for the development of novel OA strategies.

## 4. Materials and Methods

### 4.1. Patients

Adipose synovial tissue was obtained from patients with OA during knee joint replacement surgery. The experimental cohort included 32 patients (characteristics of patients in Table 1). Patients were informed about the purpose of the study and gave written consent. A non-selective beta blocker medication, targeting not only β1- but also the β2-AR, was a criterion for exclusion in this study. The project was approved by the Ethics Committee of the University of Regensburg (Ethikkommission der Medizinischen Fakultät der Universität Regensburg, vote number/project ID 13-101-0135, approved: 26 August 2015) and of the Ethics Committee Goethe University Frankfurt am Main (Ethik-Kommission des Fachbereichs Medizin Universitätsklinikum der Goethe-Universität, vote number/project ID 148-17B, approved: 10 May2017). All experiments were performed in accordance with relevant guidelines and regulations.

### 4.2. Isolation and FACS Characterization of Human ASCs

Human sASCs were isolated as described previously [42]. The cells were seeded in 75 cm^2^ tissue culture flasks and cultivated in Dulbecco’s modified Eagle medium (DMEM/F12; Gibco Invitrogen, Thermo Fisher Scientific, Darmstadt, Germany) containing 1% penicillin/streptomycin (Gibco Invitrogen) and 10% MSC qualified FBS (Gibco Invitrogen) at 37 °C in a humidified atmosphere containing 2% O_2_ and 5% CO_2_. The MSC characteristics of isolated sASCs were investigated by FACS analysis according to the suggestions of “The Mesenchymal and Tissue Stem Cell Committee of the International Society for Cellular Therapy” [34].

### 4.3. In Vitro Proliferation of sASC

Human synovial sASCs were seeded at a cell number of 2 × 10^5^ in a 75 cm^2^ flask. In addition to untreated control, cells were treated for 7 days with NE at different concentrations (10^−9^–10^−6^ M, Sigma Aldrich, Munich, Germany) at 37 °C in a humidified atmosphere containing 2% O_2_ and 5% CO_2_. NE was freshly added at day 0, 3, and 6. After seven days, total viable and dead cell number was determined.

### 4.4. Determination of Cell Viability

To determine possible toxic effects of treatments lactate dehydrogenase release (LDH Cytotoxicity Detection Kit; TaKara MK401, Shiga, Japan) was measured in supernatants of monolayer cell cultures at day 7. In addition, LDH in supernatants during chondrogenesis at day 1, 7, 14, and 21 was analyzed. Cells lysed with 1% Triton X-100 were taken as positive control and medium without cells as negative control.

### 4.5. Chondrogenic Differentiation of sASCs

In vitro chondrogenesis was performed as described earlier [18,43] by using serum-free high glucose DMEM containing 1% P/S, 100 nM dexamethasone, 200 µM ascorbate-2-phosphate, 10 ng/mL TGF-β3, 10 ng/mL BMP-6 and ITS+3 premix (Sigma). Pellets (200.000 cells per pellet) were formed by centrifugation (491× *g*) in 96-well plates with conical bottom (Nunc/Fisher Scientific, Schwerte, Germany) and were cultivated for 21 days at 37 °C in a humidified atmosphere containing and 2% O_2_ and 5% CO_2_. Pellets were treated with NE (10^−9^–10^−6^ M, Sigma). In addition, pellets were treated with specific α1-AR antagonist doxazosin (10^−7^ M, Tocris Bioscience, Bristol, UK), specific α2-AR antagonist yohimbine (10^−6^ M, Tocris Bioscience), and specific β2-AR antagonist propranolol (10^−6^ M, Tocris Bioscience, Bristol, UK), alone or in combination with NE (10^−6^ M). The differentiation medium with freshly diluted supplements was changed every two days.

### 4.6. Norepinephrine Quantification

The stability of NE at the cell culture conditions used was examined by adding a concentration of 10^−6^ M NE to the culture medium at time point zero and high-pressure liquid chromatography of medium samples after 24 h as previously described by us [44].

### 4.7. Western Blot Analysis

In order to examine whether monolayer chondrocytes respond to NE, the two major AR-dependent signaling pathways—the phosphorylation of PKA and ERK1/2—were investigated. The sASCs were treated with NE (10^−9^–10^−6^ M) and/or with specific AR antagonists as described previously [25]. ASCs were lysed and pellets were mechanically homogenized using Polytron PT-1200 (Kinematica, Thermo Fisher Scientific, Darmstadt, Germany) homogenizer and proteins were isolated using PhosphoSafe™ Extraction Reagent (Merck Millipore, Darmstadt, Germany). Samples were loaded onto 10% SDS-PAGE and electro-transferred to a polyvinylidene difluoride (PVDF) membrane. Membranes were blocked with 5% bovine serum albumin for 1 h at room temperature before incubation with primary antibodies detecting total ERK1/2 (#9107, mouse, Cell Signaling Technology, Frankfurt am Main, Germany), phosphorylated ERK1/2 (#4370; rabbit, Cell Signaling Technology), total PKA (ab32514, rabbit, Abcam, Cambridge, UK), phosphorylated PKA (ab32390, rabbit, Abcam) and GAPDH (MA5-15738, mouse, Thermo Fisher Scientific, Darmstadt, Germany) at 4 °C overnight. The membranes were incubated with a HRP-conjugated secondary antibody (swine anti-rabbit P039901-2, rabbit anti-mouse P026002-2, both from DAKO, Agilent Technologies, Hamburg.Germany) for 1 h at room temperature. The target protein was detected using the enhanced chemiluminescence (ECL, Thermo Fisher Scientific, Darmstadt, Germany) reagents, with GAPDH as endogenous control. Densitometric values of the detected bands were quantified using the ImageJ Software (https://imagej.nih.gov/ij/download.html).

### 4.8. RNA Isolation, Endpoint and Real-Time Quantitative PCR

Monolayer sASCs were lysed and pellets were homogenized mechanically (Polytron PT-1200, Kinematica). RNA isolation was performed using the NucleoSpin RNA kit (Machrey Nagel, Düren, Germany) according to the manufacturer’s instructions. cDNA synthesis was carried out using qScript cDNA Supermix (Quanta Biosciences, Beverly, MA, USA). Gene expression of α- (α1A, α1B, α1D, α2A, α2B, α2C), β-AR (β1, β2, β3) subtypes and TH was determined by qPCR using Taq PCR Master Mix kit (Qiagen, Hilden, Germany). The PCR products were run on a 1.8% (*wt*/*vol*) agarose gel, stained with GelRed Nucleic Acid Gel Stain (Biotium, Fremont, CA, USA). Average score of AR and TH gene expression in sASCs from three different OA patients was calculated. In order to analyze the effects of NE and specific antagonists on chondrogenic differentiation of sASCs, real-time quantitative PCR was performed using Quanta PerfeCta SYBR Green FastMix (Quanta Biosciences) in a qTOWER^3^ real time PCR Thermocycler (Analaytik, Jena, Jena, Germany). Gene expression of chondrogenic markers (SOX9, COL2A1), of fibrous cartilage markers (COL1A1), and hypertrophic markers (RUNX2,COL10A1, and MMP13) was quantified. GAPDH served as endogenous control. Relative gene expression was determined using qPCR software (Analytic, Jena). All primers were synthesized by Thermo Fisher Scientific (Supplementary Table S1).

### 4.9. Macroscopic and Histological Investigations

Macroscopic images of day 7, 14, and 21 pellets were taken using a standard binocular with Polaroid PDMC-3 camera. Surface areas of spherical pellets were analyzed (ImageJ software) and the pellets volume was calculated mathematically using the average radius. Then, pellets were fixed after day 7, 14, and 21 in 4% paraformaldehyde and infiltrated with increasing concentrations (10, 20, and 30%) of sucrose, each concentration for one day. Pellets were embedded in Tissue-Tek (Sakura, Alphen aan den Rijn, Netherlands) and sectioned at 8 µm thickness using a cryotom ( Cryostar NX70, Thermo Fisher Scientific, Darmstadt, Germany). Cryosections were stained with dimethylmethylene blue (DMMB, Sigma Aldrich, Munich) to visualize the sulfated glycosaminoglycans (sGAGs).

### 4.10. Immunohistochemistry

After 5 min rehydration in 1× PBS, cryosections to be stained for adrenergic receptors were demasked using citrate buffer (10 mM sodium citrate, 0.05% tween 20, pH 6) for 20 min at 95 °C. Sections for type II collagen staining were digested with 1 mg/mL pepsin in 1× McIlvaine buffer (pH 3.6) for 12–15 min at 37 °C. Then endogenous alkaline phosphatase (Bloxall, Vector Labs, Linaris, Dossenheim, Germany, 10 min, room temparature) peroxidase (0.3% H_2_O_2_ 10 min, room temperature) were blocked. Non-specific binding sites were blocked using secondary antibody-specific sera (VECTASTAIN® ABC-AP Staining KIT, Vecor Labs, Linaris, Dossenheim, Germany or HRP-AEC Kit, Linaris, Dossenheim, Germany) for 45 min at room temperature. Sections were then incubated with the primary rabbit antibodies directed against α2a AR (1:200; ab85570), β2-AR (1:200; ab213651), and type II collagen (1:200, ab34712) at 4 °C overnight. Specific staining was visualized using horseradish peroxidase labelled secondary antibodies and ALP or peroxidase substrate solutions (VECTASTAIN® ABC-AP Staining KIT, Vecor Labs, Linaris, Dossenheim, Germany or HRP-AEC Kit, Linaris, Dossenheim, Germany). 

### 4.11. Biochemical Analysis of sGAGs and Type II Collagen Protein

For the quantification of sGAGs and type II collagen levels as well as double-stranded DNA (dsDNA), pellets were mechanically homogenized using a Polytron PT-1200 (Kinematica) homogenizer and digested as described previously [18]. Concentration of dsDNA was determined using the Quant-iT PicoGreen assay kit (Invitrogen). The sGAG content of digested pellets was measured using a colorimetric assay based on dimethylmethylene blue (DMMB, Sigma) [18,42]. Type II collagen protein levels were quantified by ELISA (Chondrex, AMS Biotechnology (Europe) Ltd, Abingdon OX14 4SE, UK). sGAG and type II collagen levels were normalized to the dsDNA content.

### 4.12. Statistical Analysis

Statistical analysis was performed using SigmaPlot software (SigmaPlot V.13, Systat Software, Erkrath, Germany). All experiments were carried out with cells of 4–10 patients. Comparisons between groups were performed using ANOVA on ranks or Wilcoxon/Mann–Whitney-Test followed by Bonferroni or Dunn’s correction. *p* values less than 0.05 were considered significant.

## Figures and Tables

**Table 1:** Characteristics and medication of patients under study.

Characteristics	Number/Mean/(%)/(Range)
total (number)	32
female/male (number, %)	12/20 (37.5%/62.5%)
age (years—mean ± stdd., (range))	65.92 ± 9.84 (46–88)
C-reactive protein (mg/L)	1.48 ± 1.11
medication	
non-steroidal antiinflammatory drugs	31 (97%)
steroids (other than prednisolone)	n.a.
opioid analgesics	2 (6.25%)
biologicals	n.a.
antihypertensive drugs	19 (59.3%)
non-selective beta blockers (β1 and β2)	0 (0%)
selective beta blockers (β1)	8 (25%)
other (AT1, ACE, CCB)	11 (34.4%)

Abbreviation: n.a.—not applicable, stdd.—standard deviation, AT1—angiotensin II type 1 receptor blockers, ACE—Angiotensin converting enzyme (ACE) inhibitors, CCB—calcium channel blockers.

## Supplementary Materials

Supplementary Materials can be found at https://www.mdpi.com/1422-0067/20/13/3127/s1.

## Abbreviations

ACE	Angiotensin converting enzyme
AEC	3-Amino-9-ethylcarbazole
AP	Alkaline phosphatase
AR	Adrenoceptor
AT1	Angiotensin II type 1 receptor
BMP-6	Bone morphogenetic protein
BMSC	Bone marrow-derived MSCs
cAMP	Cyclic adenosine monophosphate
CCB	Calcium channel blockers
CD	Cluster of differentiation
cDNA	Complementary desoxy ribonucleic acid
COL10A1	Collagen type X alpha 1 chain
COL1A1	Collagen type I alpha 1 chain
COL2A1	Collagen type II alpha 1 chain
DA	Dopamine
DMEM/F12	Dulbecco’s modified eagle’s medium and Ham’s F-12 Medium
DMMB	1,9-Dimethyl-methylene blue
DPBS	Dulbeccos phosphate-buffered saline
dsDNA	Double stranded DNA
E	Epinephrine
ECM	Extracellular matrix
ELISA	Enzyme-linked immunosorbent assay
ERK	Extracellular signal-regulated kinases
FBS	Fetal bovine serum
GAG	Glycosaminoglycan
GAPDH	Glyceraldehyde-3-phosphate dehydrogenase
HLA-DR	Human Leukocyte Antigen—DR isotype
HRP	Horseradish peroxidase
ITS	Insulin, human transferrin, and selenous acid
LDH	Lactate dehydrogenase
MMP13	Matrix metallopeptidase 13
mRNA	Messenger ribonucleic acid
MSC	Mesenchymal stem cells
NE	Norepinephrine
OA	Osteoarthritis
P/S	Penicillin streptomycin
PCR	Polymerase chain reaction
PFA	Paraformaldehyde
PKA	Proteinkinase A
PVDF	Polyvinylidene difluoride
RT-PCR	Reverse-transcriptase PCR
RUNX2	Runt-related transcription factor 2
sASC	Synovial adipose stem cell
SDS-Page	Sodium dodecyl sulfate polyacrylamide gel electrophoresis
sGAG	Sulphated Glycosaminoglycans
SOX9	SRY-box 9
TBST	Tris buffered saline with Tween20
TGF-β	Transforming growth factor β
TH	Tyrosine hydroxylase

## References

[1] Pereira D., Peleteiro B., Araujo J., Branco J., Santos R.A., Ramos E. (2011). The effect of osteoarthritis definition on prevalence and incidence estimates: A systematic review. Osteoarthr. Cartil..

[2] Hoy D.G., Smith E., Cross M., Sanchez-Riera L., Blyth F.M., Buchbinder R., Woolf A.D., Driscoll T., Brooks P., March L.M. (2015). Reflecting on the global burden of musculoskeletal conditions: Lessons learnt from the global burden of disease 2010 study and the next steps forward. Ann. Rheum. Dis..

[3] Litwic A., Edwards M.H., Dennison E.M., Cooper C. (2013). Epidemiology and burden of osteoarthritis. Br. Med Bull..

[4] Loeser R.F., Goldring S.R., Scanzello C.R., Goldring M.B. (2012). Osteoarthritis: A disease of the joint as an organ. Arthritis Rheum..

[5] Martel-Pelletier J., Barr A.J., Cicuttini F.M., Conaghan P.G., Cooper C., Goldring M.B., Goldring S.R., Jones G., Teichtahl A.J., Pelletier J.P. (2016). Osteoarthritis. Nat. Reviews. Dis. Primers.

[6] McGonagle D., Tan A.L., Carey J., Benjamin M. (2010). The anatomical basis for a novel classification of osteoarthritis and allied disorders. J. Anat..

[7] Mobasheri A., Kalamegam G., Musumeci G., Batt M.E. (2014). Chondrocyte and mesenchymal stem cell-based therapies for cartilage repair in osteoarthritis and related orthopaedic conditions. Maturitas.

[8] De Bari C., Dell’Accio F., Tylzanowski P., Luyten F.P. (2001). Multipotent mesenchymal stem cells from adult human synovial membrane. Arthritis Rheum..

[9] McGonagle D., Baboolal T.G., Jones E. (2017). Native joint-resident mesenchymal stem cells for cartilage repair in osteoarthritis. Nat. Reviews. Rheumatol..

[10] Murphy J.M., Dixon K., Beck S., Fabian D., Feldman A., Barry F. (2002). Reduced chondrogenic and adipogenic activity of mesenchymal stem cells from patients with advanced osteoarthritis. Arthritis Rheum..

[11] Sekiya I., Muneta T., Horie M., Koga H. (2015). Arthroscopic transplantation of synovial stem cells improves clinical outcomes in knees with cartilage defects. Clin. Orthop. Relat. Res..

[12] Grässel S.G. (2014). The role of peripheral nerve fibers and their neurotransmitters in cartilage and bone physiology and pathophysiology. Arthritis Res..

[13] Jones K.B., Mollano A.V., Morcuende J.A., Cooper R.R., Saltzman C.L. (2004). Bone and brain: A review of neural, hormonal, and musculoskeletal connections. Iowa Orthop. J..

[14] Maestroni G.J. (2000). Neurohormones and catecholamines as functional components of the bone marrow microenvironment. Ann. New York Acad. Sci..

[15] Bjurholm A., Kreicbergs A., Terenius L., Goldstein M., Schultzberg M. (1988). Neuropeptide y-, tyrosine hydroxylase- and vasoactive intestinal polypeptide-immunoreactive nerves in bone and surrounding tissues. J. Auton. Nerv. Syst..

[16] Miller L.E., Justen H.P., Scholmerich J., Straub R.H. (2000). The loss of sympathetic nerve fibers in the synovial tissue of patients with rheumatoid arthritis is accompanied by increased norepinephrine release from synovial macrophages. Faseb J..

[17] Pongratz G., Straub R.H. (2013). Role of peripheral nerve fibres in acute and chronic inflammation in arthritis. Nat. Reviews. Rheumatol..

[18] Jenei-Lanzl Z., Grässel S., Pongratz G., Kees F., Miosge N., Angele P., Straub R.H. (2014). Norepinephrine inhibition of mesenchymal stem cell and chondrogenic progenitor cell chondrogenesis and acceleration of chondrogenic hypertrophy. Arthritis Rheumatol.

[19] Pongratz G., Straub R.H. (2014). The sympathetic nervous response in inflammation. Arthritis Res. Ther..

[20] Molinoff P.B. (1984). Alpha- and beta-adrenergic receptor subtypes properties, distribution and regulation. Drugs.

[21] Venkatakrishnan A.J., Deupi X., Lebon G., Tate C.G., Schertler G.F., Babu M.M. (2013). Molecular signatures of g-protein-coupled receptors. Nature.

[22] Blesen T.v., Hawes B.E., Luttrell D.K., Krueger K.M., Touhara K., Porfflri E., Sakaue M., Luttrell L.M., Lefkowitz R.J. (1995). Receptor-tyrosine-kinase- and gβγ-mediated map kinase activation by a common signalling pathway. Nature.

[23] Bogoyevitch M.A., Andersson M.B., Gillespie-Brown J., Clerk A., Glennon P.E., Fuller S.J., Sugden P.H. (1996). Adrenergic receptor stimulation of the mitogen-activated protein kinase cascade and cardiac hypertrophy. Biochem. J..

[24] Alblas J., van Corven E.J., Hordijk P.L., Milligan G., Moolenaar W.H. (1993). Gi-mediated activation of the p21ras-mitogen-activated protein kinase pathway by alpha 2-adrenergic receptors expressed in fibroblasts. J. Biol. Chem..

[25] Jiao K., Zeng G., Niu L.N., Yang H.X., Ren G.T., Xu X.Y., Li F.F., Tay F.R., Wang M.Q. (2016). Activation of alpha2a-adrenergic signal transduction in chondrocytes promotes degenerative remodelling of temporomandibular joint. Sci. Rep..

[26] Lorenz J., Schafer N., Bauer R., Jenei-Lanzl Z., Springorum R.H., Grassel S. (2016). Norepinephrine modulates osteoarthritic chondrocyte metabolism and inflammatory responses. Osteoarthr. Cartil..

[27] Mitchell J., Lai L.P., Peralta F., Xu Y., Sugamori K. (2011). Beta2-adrenergic receptors inhibit the expression of collagen type ii in growth plate chondrocytes by stimulating the ap-1 factor jun-b. Am. J. Physiology. Endocrinol. Metab..

[28] Lafont J.E. (2010). Lack of oxygen in articular cartilage: Consequences for chondrocyte biology. Int. J. Exp. Pathol..

[29] Pattappa G., Johnstone B., Zellner J., Docheva D., Angele P. (2019). The importance of physioxia in mesenchymal stem cell chondrogenesis and the mechanisms controlling its response. Int. J. Mol. Sci..

[30] Mandl L.A. (2019). Osteoarthritis year in review 2018: Clinical. Osteoarthr. Cartil..

[31] Huang Y.Z., Xie H.Q., Silini A., Parolini O., Zhang Y., Deng L., Huang Y.C. (2017). Mesenchymal stem/progenitor cells derived from articular cartilage, synovial membrane and synovial fluid for cartilage regeneration: Current status and future perspectives. Stem Cell Rev..

[32] Grogan S.P., Miyaki S., Asahara H., D’Lima D.D., Lotz M.K. (2009). Mesenchymal progenitor cell markers in human articular cartilage: Normal distribution and changes in osteoarthritis. Arthritis Res..

[33] Capellino S., Cosentino M., Wolff C., Schmidt M., Grifka J., Straub R.H. (2010). Catecholamine-producing cells in the synovial tissue during arthritis: Modulation of sympathetic neurotransmitters as new therapeutic target. Ann. Rheum. Dis..

[34] Dominici M., Le Blanc K., Mueller I., Slaper-Cortenbach I., Marini F., Krause D., Deans R., Keating A., Prockop D., Horwitz E. (2006). Minimal criteria for defining multipotent mesenchymal stromal cells. The international society for cellular therapy position statement. Cytotherapy.

[35] Kotova P.D., Sysoeva V.Y., Rogachevskaja O.A., Bystrova M.F., Kolesnikova A.S., Tyurin-Kuzmin P.A., Fadeeva J.I., Tkachuk V.A., Kolesnikov S.S. (2014). Functional expression of adrenoreceptors in mesenchymal stromal cells derived from the human adipose tissue. Biochim. Et Biophys. Acta.

[36] Tyurin-Kuzmin P.A., Fadeeva J.I., Kanareikina M.A., Kalinina N.I., Sysoeva V.Y., Dyikanov D.T., Stambolsky D.V., Tkachuk V.A. (2016). Activation of beta-adrenergic receptors is required for elevated alpha1a-adrenoreceptors expression and signaling in mesenchymal stromal cells. Sci. Rep..

[37] Speichert S., Molotkov N., El Bagdadi K., Meurer A., Zaucke F., Jenei-Lanzl Z. (2019). Role of norepinephrine in il-1beta-induced chondrocyte dedifferentiation under physioxia. Int. J. Mol. Sci..

[38] Sheng G. (2015). The developmental basis of mesenchymal stem/stromal cells (mscs). Bmc Dev. Biol..

[39] Jenei-Lanzl Z., Meurer A., Zaucke F. (2019). Interleukin-1beta signaling in osteoarthritis - chondrocytes in focus. Cell. Signal..

[40] Anderson D.E., Markway B.D., Weekes K.J., McCarthy H.E., Johnstone B. (2018). Physioxia promotes the articular chondrocyte-like phenotype in human chondroprogenitor-derived self-organized tissue. Tissue Engineering. Part. A.

[41] Jenei-Lanzl Z., Zwingenberg J., Lowin T., Anders S., Straub R.H. (2015). Proinflammatory receptor switch from galphas to galphai signaling by beta-arrestin-mediated pde4 recruitment in mixed ra synovial cells. Brain Behav. Immun..

[42] Jenei-Lanzl Z., Straub R.H., Dienstknecht T., Huber M., Hager M., Grassel S., Kujat R., Angele M.K., Nerlich M., Angele P. (2010). Estradiol inhibits chondrogenic differentiation of mesenchymal stem cells via nonclassic signaling. Arthritis Rheum..

[43] Hennig T., Lorenz H., Thiel A., Goetzke K., Dickhut A., Geiger F., Richter W. (2007). Reduced chondrogenic potential of adipose tissue derived stromal cells correlates with an altered tgfbeta receptor and bmp profile and is overcome by bmp-6. J. Cell. Physiol..

[44] Kees M.G., Pongratz G., Kees F., Scholmerich J., Straub R.H. (2003). Via beta-adrenoceptors, stimulation of extrasplenic sympathetic nerve fibers inhibits lipopolysaccharide-induced tnf secretion in perfused rat spleen. J. Neuroimmunol..

