# Supplementary material for: Norepinephrine Inhibits Synovial Adipose Stem Cell Chondrogenesis via α2a-Adrenoceptor-Mediated ERK1/2 Activation
Source: Int J Mol Sci. 2019 Jun 26;20(13):3127. doi: 10.3390/ijms20133127 (PMC6651223; doi:10.3390/ijms20133127)
Supplement: Supplementary file 1 [file ijms-20-03127-s001.pdf]

International Journal of
Molecular Sciences

MDPI

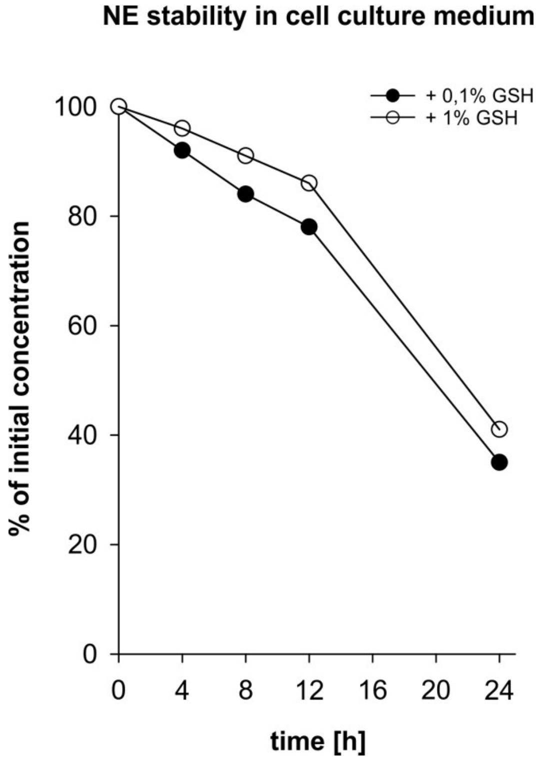

Figure S1. Stability of NE in cell culture medium over 24 h in the presence of the antioxidant glutathione (GSH 0.1% or 1%).

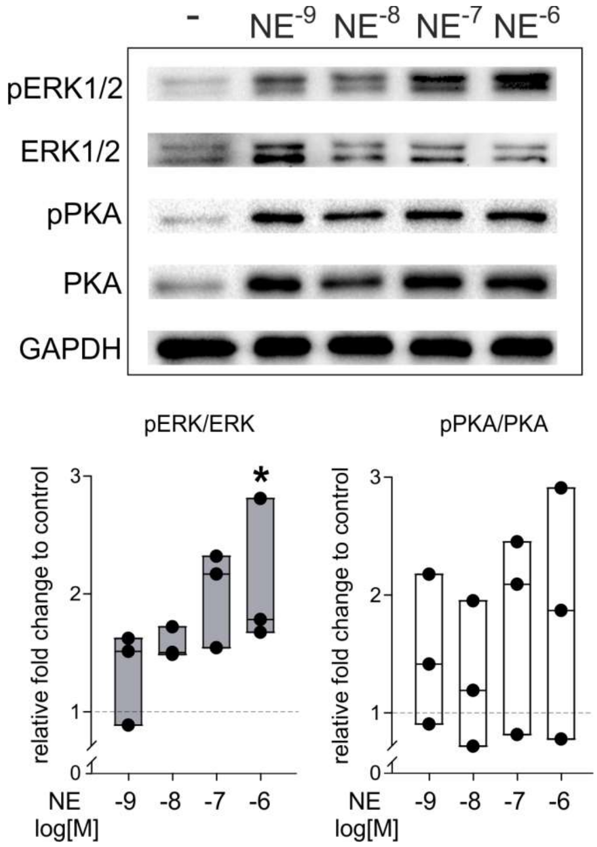

Figure S2. NE-mediated intracellular signaling. Expression of ERK1/2 and PKA as well as dose-dependent phosphorylation of ERK1/2 in sASC culture.

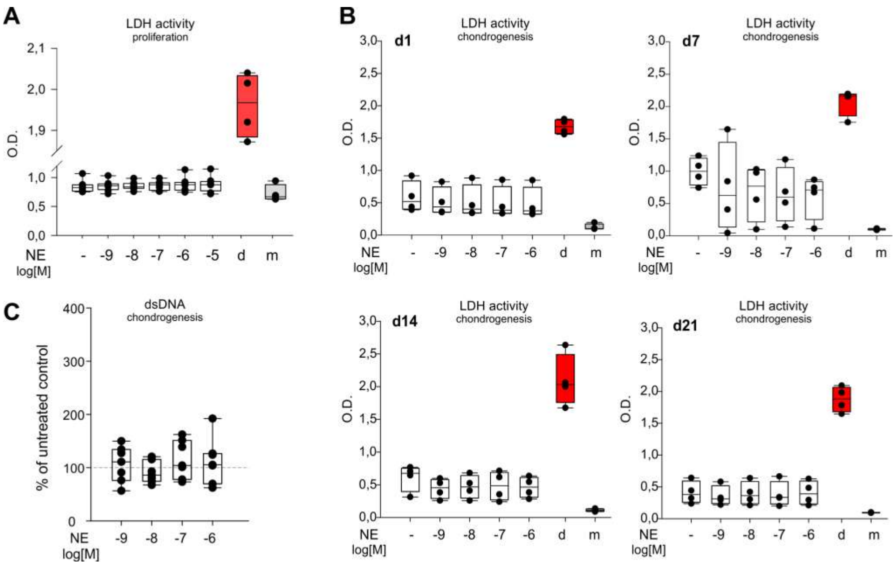

Figure S3. Effect of NE on sASC viability and dsDNA content (A) LDH release in monolayer sASCs in the presence of NE in different concentrations (10–9-10–5 M). (B) LDH release in untreated and NE-treated (10–9-10–5 M) three-dimensional chondrogenic pellet culture at day 1, 7, 14 and 21 of differentiation. C) Biochemical quantification of dsDNA content in untreated and NE-treated (10–9-10–5 M) three-dimensional chondrogenic pellet culture at day 21 of differentiation. Values are shown in percent of untreated control (= 100%, dashed line). Each black circle represents an individual patient. Box plots are explained in legend to Figure 1. Abbreviations: d – dead control (positive control), m – medium (negative control).

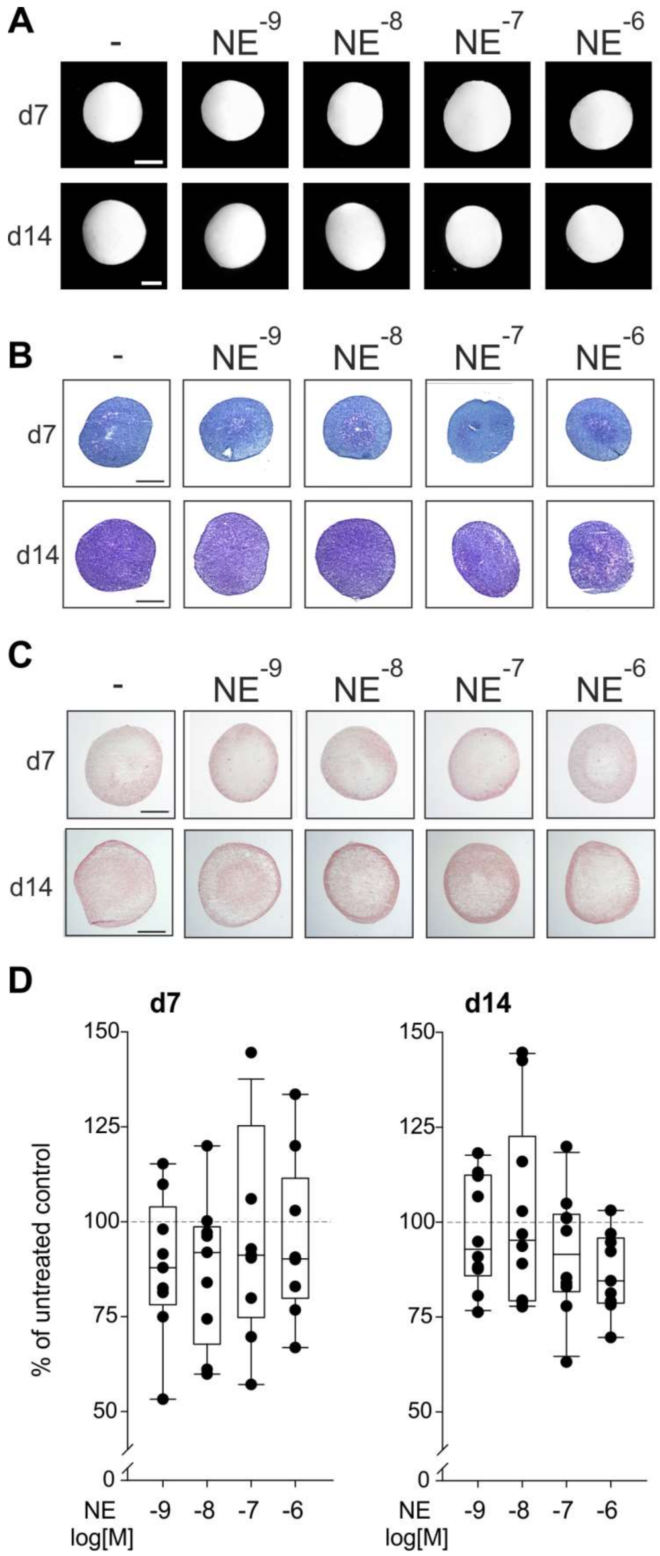

Figure S4. Macroscopic, histologic (sGAG) analysis and immunohistochemical type II collagen l staining of untreated or NE-treated (10–9-10–6 M) chondrogenic sASC pellets at d7 and d14 of differentiation (bars, 500 µm).

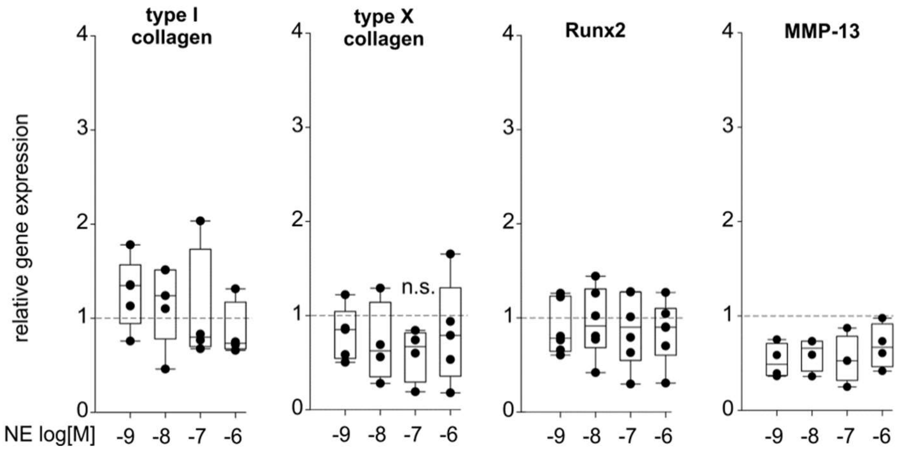

Figure S5. Effect of NE on the expression of fibrous cartilage and hypertrophic marker genes during sASC chondrogenesis. (A) Expression of COL1A1, COL10A1, RUNX2, and MMP13 in untreated and NE-treated (10^{-9}–10^{-6} M) chondrogenic sASC pellets at day 21. Each circle shows the mean of 3 replicates per patient (*n* = 4–6). Values are fold expression of control, where the control = 1 (represented by the dashed line). Box plots are explained in legend to Figure 1.

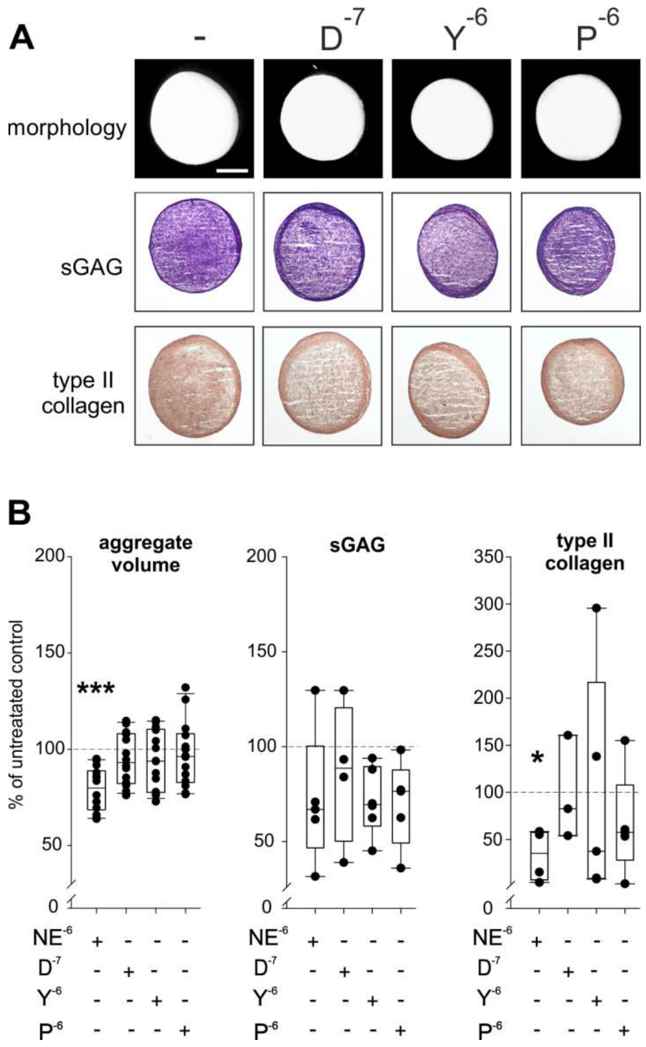

Figure S6. Effect of specific AR antagonists during sASC chondrogenesis. (A) Macroscopic, histologic (sGAG) analysis and immunohistochemical type II collagen staining of chondrogenic sASC pellets treated with specific AR antagonists at day 21 of differentiation (bars 500 μm). (B) Quantification of pellet volume and biochemical quantification of type II collagen and sGAG content of chondrogenic

sASC pellets treated with specific AR antagonists at day 21. Each circle shows the mean of 3 replicates per patient (*n* = 4–6). Values are the percent of control (control =100%, dashed line). Box plots are explained in legend to Figure 1. Significant *p*-values against untreated control are presented as * $p < 0.05$, ** $p < 0.01$, *** $p < 0.001$. Abbreviations: D – doxazosin, Y – yohimbine, P – propranolol.

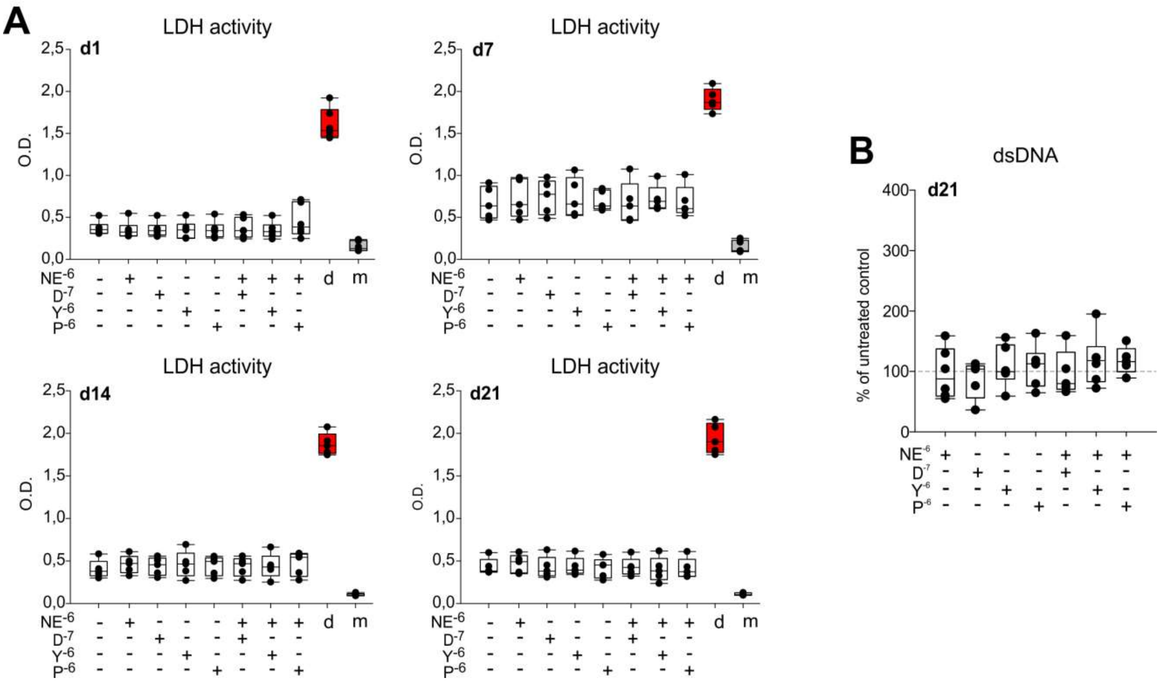

Figure S7. Effect of specific antagonists on sASC viability and dsDNA content (A) LDH release in three-dimensional chondrogenic sASC pellets treated with NE (10–6 M) or with NE plus specific AR antagonists at day 1, 7, 14 and 21 of differentiation. B) Biochemical quantification of dsDNA content in in three-dimensional chondrogenic sASC pellets treated with NE (10–6 M) or with NE plus specific AR antagonists at day 21 of differentiation. Values are demonstrated in percent of the control (untreated control = 100%, dashed line). Each black circle represents an individual patient. Box plots are explained in legend to Figure 1. Abbreviations: D – doxazosin, Y – yohimbine, P – propranolol, d – dead control (positive control), m – medium (negative control).

Table S1. The primer sequences used for PCR.

Gene Name	NCBI Reference	Forward (5′-3′)	Reverse (5′-3′)
GAPDH	NM_001289745.2	CTCCTGTTCGACAGTCAGCC	TTCCCGTTCTCAGCCTTGAC
TH	NM_000360.3	CAGGCAGAGGCCATCATGT	GTGGTCCAAGTCCAGGTCAG
ADRA1A	NM_000680.3	CCATGCTCCAGCCAAGAGTT	TCCTGTCCTAGACTTCCTCCC
ADRA1B	NM_000679.3	GTCCACCGTCATCTCCATCG	GAACAAGGAGCCAAGCGGTAG
ADRA1D	NM_000678.3	TGACTTTCCGCGATCTCCTG	TTACCTGCCACGGCCATAAG
ADRA2A	NM_000681.3	TGGTCATCGGAGTGTTCGTG	GCCCACTAGGAAGATGGCTC
ADRA2B	NM_000682.6	GACATTTCACCGGCAACACC	GGGACTGAGAACCAGGAAGC
ADRA2C	NM_000683.3	CGATGTGCTGTTTTGCACCT	GGATGTACCAGGTCTCGTCG

ADRB1	NM_000684.2	TAGCAGGTGAACTCGAAGCC	ATCTTCCACTCCGGTCCTCT
ADRB2	NM_000024.5	CAGAGCCTGCTGACCAAGAA	GCCTAACGTCTTGAGGGCTT
ADRB3	NM_000025.3	GCCAATTCTGCCTTCAACCC	GCCAGAGGTTTTCCACAGGT
COL1A1	NM_000088.3	ACGTCCTGGTGAAGTTGGTC	ACCAGGGAAGCCTCTCTCTC
COL2A1	NM_001844.4	TTCAGCTATGGAGATGACAATC	AGAGTCCTAGAGTGACTGAG
MMP13	NM_002427.4	GACTGGTAATGGCATCAAGGGA	CACCGGCAAAAGCCACTTTA
COL10A1	XM_011535433.3	CCCTCTTGTTAGTGCCAACC	AGATTCCAGTCCTTGGGTCA
RUNX2	XM_011514966.2	GGAGTGGACGAGGCAAGAGTTT	AGCTTCTGTCTGTGCCTTCTGG
SOX9	NM_000346.4	ACACACAGCTCACTCGACCTTG	AGGGAATTCTGGTTGCTCCTCT
